# Estimating the prevalence, socioeconomic determinants, and health seeking behavior of individuals with depression in Ghana

**DOI:** 10.1038/s41598-025-06134-2

**Published:** 2025-07-01

**Authors:** Michel Adurayi Amenah, Ama Fenny, James Akazili, Tolib Mirzoev, Irene Akua Agyepong, Thomas Mason

**Affiliations:** 1 NIHR Global Health Research Centre for Non-communicable Disease Control in West Africa, Ghana College of Physicians and Surgeons, Accra, Ghana; 2 Division of Health Research, Lancaster University, Lancaster, UK; 3 Institute of Statistical Social and Economic Research, University of Ghana, Accra, Ghana; 4 School of Public Health, C.K. Tedam University of Technology and Applied Science, Navrongo, Ghana; 5 Bergen Centre for Ethics and Priority, University of Bergen, Bergen, Norway; 6 Department of Global Health and Development, London School of Hygiene and Tropical Medicine, London, UK

**Keywords:** Mental health, Ghana, Depression, Health-seeking behaviour, Socioeconomic factors, Diseases, Health care

## Abstract

Depression is a growing public health concern in Ghana, exacerbated by limited mental health services, stigma, and socio-economic disparities. While awareness is rising, research on its prevalence, health-seeking behavior, and socio-economic determinants remains limited. This study estimates depression prevalence, assesses health-seeking behavior, and identifies socio-economic drivers influencing these outcomes. Using three waves of the Ghana Socioeconomic Panel Survey (2009/2010, 2013/2014, 2018/2019), depression was measured via the Kessler Psychological Distress Scale (K10). Logistic regression and random-effects models analyzed the socio-economic factors affecting depression and mental health-seeking behavior. Depression prevalence declined from 31.04% in 2009/2010 to 27.21% in 2013/2014 but rose again to 29.54% in 2018/2019. Health-seeking behavior improved from 6.38 to 14.88%, though rural and disadvantaged groups remained underserved. Older adults (65+) and Northern Region residents had significantly higher odds of depression, while post-secondary education reduced risk. Unemployment correlated with lower depression odds, likely reflecting the protective role of communal networks in Ghana. Depression remains a significant issue, with persistent disparities in access to care. Expanding rural mental health services, improving mental health literacy, and addressing regional inequalities are crucial to reducing Ghana’s mental health burden.

**Supplementary Information:**

The online version contains supplementary material available at 10.1038/s41598-025-06134-2.

## Introduction

Mental health is an integral aspect of overall well-being, yet it remains one of the most overlooked components of healthcare systems worldwide. Globally, mental health disorders, particularly depression, are significant contributors to the burden of disease, affecting millions of individuals and communities^1,2^ and standing as the leading cause of disability by impacting approximately 5% of the adult population, contributing substantially to lost productivity and diminished quality of life^3^. Despite this profound impact, mental health continues to be underfunded, particularly in low- and middle-income countries (LMICs), where structural inequities, resource constraints, stigma, insufficient resources, and weak healthcare infrastructure exacerbate the treatment gap^3,4^. Approximately 1 in 8 individuals is affected by a mental health condition, yet up to 85% of those in LMICs do not receive adequate care^4,6^. Even in high-income countries, mental health systems struggle to meet demand due to chronic underfunding and service fragmentation^5^, and these challenges are compounded in LMICs by severe shortages of mental health professionals and limited integration of mental health into primary healthcare^6^. While global initiatives such as the World Health Organization (WHO)’s Mental Health Action Plan (2013–2030) aim to promote community-based mental health care and universal coverage, progress has been slow and uneven, leaving vulnerable populations disproportionately affected^7^.

In Ghana, the mental health landscape mirrors global challenges, with inadequate services further strained by stigma and limited infrastructure^8,9^. The Mental Health Authority, established under the Mental Health Act 846, has made strides in integrating mental health into primary healthcare^10^. However, these efforts are significantly hindered by resource limitations. Mental health receives less than 1.4% of Ghana’s total health budget, reflecting a long-standing neglect of mental health within national health priorities^11^. Furthermore, over 80% of the country’s psychiatric professionals are concentrated in just three urban psychiatric hospitals; Accra, Pantang, and Ankaful, leaving most rural districts without dedicated personnel or services^12–14^. This urban–rural imbalance contributes to stark inequities in access, particularly in northern and remote regions^6^. Despite these challenges, mental health is increasingly recognized as a critical public health priority, underscoring the need for targeted policies and interventions to address the growing burden^15^.

Estimates of depression prevalence in Ghana vary widely depending on the population studied, geographic setting, and the tools used. National-level data suggest that approximately 10–12% of Ghanaian adults experience depression at a given time^16^, based on WHO World Health Survey data using ICD-10 criteria. However, regional and community studies report much higher figures, for example, 25.2% among adults in the Volta Region^17^ and 12.3% during the COVID-19 pandemic^18^. Adolescents and students show some of the highest rates, with senior high school students in northern Ghana reporting 58–69%^19^, while university students in Greater Accra report 39%^20^ and nursing students in the north show 58% moderate-to-severe depression^21^. Among older adults, estimates range from 6 to 7% based on diagnostic interviews^22^ to 42.1% using the Geriatric Depression Scale^23^. Pregnant and postpartum women also experience high rates, with systematic reviews showing 4–11% postpartum depression in southern Ghana, 17–33% in the north^24^, and 38.6% among pregnant adolescents^25^.

The social determinants of depression in Ghana reflect complex interactions between socioeconomic, cultural, and environmental factors Gender can impact mental health experiences, with women often facing higher rates of depression due to societal pressures, while cultural expectations may discourage men from seeking help^26^. Age is also critical: adolescents may encounter stress from school and social pressures, while the elderly often face isolation, increasing their vulnerability to depression^27^. Regional disparities, especially in rural areas, limit access to mental health services, leaving needs unmet^28^. Marital status plays a role, as separated or widowed individuals may experience heightened depression due to social and economic challenges, while religious beliefs can both support and stigmatize mental health care^29^. Employment status provides stability, whereas unemployment can heighten mental health risks^30^. Education affects awareness and treatment-seeking, with lower educational levels exacerbating mental health challenges^31^.

Health-seeking behavior for depression in Ghana is shaped by socio-demographic, cultural, systemic, and psychological factors. Higher education and income levels significantly increase the likelihood of seeking professional care, as they enhance awareness and affordability, while employment also plays a positive role. However, gender differences appear less pronounced, emphasizing the need for inclusive interventions^32,33^. Cultural beliefs and stigma remain substantial barriers, with traditional views attributing mental illness to supernatural causes, deterring individuals from clinical care. Stigma, both internalized and societal, exacerbates this reluctance^9,33^. Systemic challenges, including urban-centric services, financial constraints, and poor infrastructure, further hinder access, particularly in rural areas^34^. Psychological factors like locus of control and strong social support networks positively influence health-seeking behaviors by empowering individuals and reducing isolation^33,35^.

While these studies consistently highlight depression as a widespread and serious public health issue across all demographic groups^16,17,20,23^, their reported prevalence figures differ based on diagnostic approach, setting, and population characteristics. Screening tools like PHQ-9, DASS-21, and CES-D often yield higher prevalence rates because they include mild and subthreshold symptoms^19,25,36^, whereas structured diagnostic interviews (ICD-10, K-SADS) report lower rates, focusing only on clinical cases^16,22^. Regional variations are also striking, with northern Ghana frequently showing higher depression rates than the south, reflecting underlying socioeconomic disparities and limited access to mental health services^24,25,36^. Moreover, facility-based studies tend to report elevated prevalence, likely because individuals seeking clinical care may represent more severe or complicated cases compared to those surveyed in the general community^24^.

Addressing depression in Ghana is crucial not only because of its high prevalence but also because of its wide-ranging personal, social, and economic impacts. Depression impairs individuals’ quality of life, reduces productivity, increases healthcare costs, and reinforces poverty and social inequality, particularly among vulnerable groups such as adolescents, pregnant women, the elderly, and the unemployed^35^. Without targeted action, these burdens ripple outward, affecting families, communities, and national development goals. Tackling depression requires multifaceted strategies: strengthening the integration of mental health into primary healthcare services, decentralizing services to reach rural and underserved areas^21^, increasing mental health funding^11^, expanding anti-stigma and mental health literacy programs^37^, and engaging traditional and community-based healers as collaborative partners^38^. Focusing on socio-economic and demographic variables such as education, income, employment, region, and gender can help policymakers design interventions that directly address the structural and cultural factors fuelling depression, ensuring more equitable and effective mental health care across Ghana.

Current evidence on depression and health-seeking behavior in Ghana, as reviewed in the preceding sections, reveals important gaps and limitations. First, most studies rely on localized or demographic-specific samples, limiting generalizability to the broader population^17,22^. Second, the heterogeneity in study methodologies and diagnostic tools results in inconsistent prevalence estimates, complicating efforts to understand the national burden^35,39^. Third, systemic and structural barriers, such as limited rural data and underexplored cultural factors, hinder the applicability of findings to underserved populations^9,34^.

Recognizing these challenges, this study seeks to address the identified gaps by using a nationally representative sample from a panel dataset collected over 10 years in three waves, providing a unique longitudinal perspective on depression and health-seeking behaviours. Its objectives are threefold: first, to estimate the prevalence of depression levels using the Kessler Psychological Distress Scale (K10); second, to assess the rate of health-seeking behavior among individuals experiencing depression; and third, to examine the socio-economic factors that influence both depression and health-seeking behaviours.

These objectives address gaps in existing literature, particularly the limited focus on nationally representative samples and comprehensive analyses of health-seeking rates specifically among those with depression. This study’s longitudinal approach allows for a richer understanding of how depression and health-seeking behaviours evolve over time, as well as the role of socio-economic factors across varied demographic contexts. The findings will enhance the current understanding of mental health support needs in Ghana, offering insights to inform policies and interventions tailored to specific socio-economic and demographic groups.

## Methods

### Data

The data for this study were drawn from three waves of the Ghana Socioeconomic Panel Survey (GSEPS), specifically the 2009/2010, 2013/2014, and 2019/2020 waves. The GSEPS used a consistent two-stage stratified sampling strategy across its waves, ensuring national and regional representation^40,41^. In Wave 1 (2009/2010), 5009 households from 334 Enumeration Areas (EAs) were selected using the 2000 Ghana Population and Housing Census. Fifteen households were randomly sampled from each EA, with smaller regions like Upper East and Upper West oversampled to enhance precision^41^. This wave provided baseline data across Ghana’s ten regions. Wave 2 (2013/2014) included 5,010 households, incorporating split or relocated households to maintain sample integrity and expanded coverage^42^. The introduction of Computer-Aided Personal Interviewing (CAPI) improved data collection efficiency. In Wave 3 (2018/2019), 5,669 households were sampled, with expanded variables including psychological and social networking data to enhance longitudinal insights. Each wave maintained a two-stage sampling method to ensure consistent national representation (ISSER, 2020). To address potential bias from oversampling, sampling weights were applied to adjust for non-self-weighting. These sampling strategies ensured that our panel remains representative of the Ghanaian population, allowing for robust longitudinal analyses across socio-economic and health dimensions.

### Variables and measurements

#### Depression

Depression is a primary focus of this study, analyzed in terms of both prevalence and severity, using the Kessler Psychological Distress Scale (K10) to assess depressive symptoms. The K10 is a validated and widely recognized tool known for its reliability in evaluating psychological distress across diverse populations^43,44^. It measures depressive symptoms over a specific timeframe through 10 items rated on a 5-point Likert scale, ranging from “None of the time” to “All of the time.“(See Supplementary Table S1). The cumulative score, ranging from 10 to 50, enables categorization of respondents by severity: No Significant Depression (0–19), Mild Depression, Moderate Depression, and Severe Depression (30 and above)^45^. K10’s ability to differentiate degrees of psychological distress offers a nuanced understanding of functional impairment, allowing for targeted insights into mental health trends across socio-demographic groups^46^.

Health-seeking behavior is the second outcome examined in this study, focusing on whether individuals experiencing depressive symptoms sought professional mental health care. This outcome is measured through self-reported responses to questions regarding actions taken to address depressive symptoms. Specifically, respondents were asked whether they had consulted healthcare professionals, such as psychiatrists, psychologists, or general practitioners. The binary outcome distinguishes between those who sought professional care (yes/no).

#### Socio-economic variables

We selected socio-economic variables for this study to provide a comprehensive understanding of the factors influencing depression and health-seeking behavior in Ghana, with a focus on their contextual relevance. These variables were chosen based on their established roles in shaping mental health outcomes, as documented in global and regional studies, and their significance within the Ghanaian socio-cultural landscape (See Supplementary Table S2).

Gender was included to capture disparities in mental health experiences and care-seeking behavior, acknowledging that societal norms often lead to higher reported depression rates among women and greater stigma around mental health care for men^47^. Age, categorized into life stages from children to elderly, reflects the unique challenges faced across the lifespan, with older adults often experiencing isolation and declining health, while younger populations face academic and social pressures^48^.

Regional analysis was critical, given the geographic disparities in mental health infrastructure, particularly between the Northern and Southern regions, where cultural and economic resources differ significantly^49^. Marital status was included to examine the protective role of social support in reducing depression, particularly for married individuals, while widowhood often increases vulnerability^50^. Religious affiliation was considered to explore its dual role in providing emotional support and, at times, perpetuating stigma^51^.

Educational level and employment status were chosen as indicators of socio-economic stability and awareness. Higher education is often associated with reduced stigma and greater mental health literacy, while employment provides the financial resources needed to seek care^52^. Lastly, communal engagement was included to assess the protective effect of social connectedness and its potential to mitigate or exacerbate mental health challenges through stigma-related barriers. These variables, carefully selected for their relevance, allowed us to capture the multifaceted nature of mental health and care-seeking behaviors in Ghana.

### Empirical models

In this study, we conduct both wave-specific cross-sectional analyses (2009, 2014, and 2019) to explore patterns within each survey round, as well as pooled longitudinal (panel) analyses using all waves combined. This dual approach allows us to capture both cross-sectional snapshots and long-term dynamics across time. The cross-sectional models are estimated separately for each wave, while the panel models leverage the full longitudinal sample to assess changes over time.

We apply both cross-sectional and panel logit regression models to examine the relationships between depression prevalence, health-seeking behavior, and socioeconomic factors. These models allow for an exploration of the temporal and cross-sectional dynamics in depression status and health-seeking behavior across survey waves^53,54^.

#### Cross-sectional logit regression model for depression status

We begin by modelling the probability of an individual being classified as depressed (K10 score ≥ 20) versus not depressed (K10 score ≤ 19) in each survey wave. The probability of $\:{Y}_{it}=1$ for individual $\:i$ in wave $\:t$ is defined as:

1 $$\:\text{Pr}\left({Y}_{it}=1\right|{X}_{it})=\:\frac{\text{exp}\left({X}_{it}\:\beta\:\right)}{1+\:\text{exp}\left({X}_{it}\:\beta\:\right)}.$$

where $\:{X}_{it}\:$includes socioeconomic factors such as age, gender, employment, marital status, and region, and $\:\beta\:$ is a vector of coefficients. To interpret the effects, we present odds ratios, which allow for understanding the relative likelihood of depression associated with each predictor. The odds ratios provide insights into the relationship between predictors and depression status, aligning with established practices in health research

#### Cross-sectional logit regression model for health seeking behavior

For each wave, we specify a logit model to analyze the probability of seeking healthcare among those experiencing depression and to explore how socioeconomic factors contribute to healthcare-seeking behavior^55^.

The probability of seeking care $\:{(Y}_{it}=1)\:$is:

2 $$\:\text{Pr}\left({Y}_{it}=1\right|{X}_{it}=\:\frac{\text{exp}\left({X}_{it}\:\beta\:\right)}{1+\:\text{exp}\left({X}_{it}\:\beta\:\right)}.$$

where $\:{Y}_{it}=1$ if individual $\:i$ in wave $\:t$ sought health care for depression. We calculate odds ratios to interpret the likelihood of seeking care associated with each socioeconomic factor, holding other variables constant.

#### Panel logit regression model for depression status

Leveraging the longitudinal nature of the data, we extend the model to a panel logit regression to control for unobserved, time-invariant individual-specific characteristics. Panel logit models are particularly valuable in accounting for unobserved heterogeneity, which may influence depression status^56,57^.

The probability that $\:{Y}_{it}=1$ is modeled as follows:

3 $$\:\text{Pr}\left({Y}_{it}=1\right|{X}_{it},\:{\alpha\:}_{i})=\:\frac{\text{exp}({X}_{it}\:\beta\:+\:{\alpha\:}_{i})}{1+\:\text{exp}({X}_{it}\:\beta\:+\:{\alpha\:}_{i})}.$$

where $\:{\alpha\:}_{i}$​ captures unobserved characteristics such as genetic predisposition or stable personal traits.

Since depression is recorded as a binary variable (1 = depressed, 0 = not depressed) and the outcome did not vary within individuals across waves, unit fixed effects could not be estimated. Consequently, random-effects (RE) estimation was used^58,59^. The RE model assumes that unobservable individual-specific characteristics ($\:{\alpha\:}_{i}$), such as genetic predispositions or stable traits, are uncorrelated with the observed predictors ($\:{X}_{it}$), attributing these effects to a specified distribution^60,61^. This approach allows for the inclusion of time-invariant variables and provides consistent estimates but relies on the strong assumption that unobserved heterogeneity is not correlated with predictors like income or education. If this assumption does not hold, the estimates may be biased, warranting caution when interpreting results^62^.

#### Panel logit regression model for health seeking behaviour

The probability of seeking healthcare for depression over time is similarly modeled with a panel logit approach:

4 $$\:\text{Pr}\left({Y}_{it}=1\right|{X}_{it},\:{\alpha\:}_{i})=\:\frac{\text{exp}({X}_{it}\:\beta\:+\:{\alpha\:}_{i})}{1+\:\text{exp}({X}_{it}\:\beta\:+\:{\alpha\:}_{i})}.$$

Where $\:{Y}_{it}=1$ if care was sought. Since health-seeking behavior also showed limited within-individual variation across waves, the fixed-effects model was not applicable, and we opted for a random-effects model. We utilized Stata 18 for all statistical analyses^63^.

To enhance statistical validity, we applied cluster-robust standard errors in all panel regression models to address potential heteroskedasticity and within-cluster correlation, ensuring reliable standard error estimates^64^. This approach accounts for the hierarchical structure of the data, particularly within households and regions, aligning with best practices in health economics and strengthening the precision and robustness of our findings.

## Results

### Socio-economic characteristics

Table 1 provides a summary of the demographic characteristics of the study population across the three survey waves (2009/2010, 2013/2014, and 2019/2020). The gender distribution remained relatively stable, with females representing approximately 52/3% of the population in each wave. In terms of age, children (0–14 years) constituted the largest age group, although their proportion decreased from 40.13% in 2009/2010 to 33.7% in 2019/2020. The proportion of younger persons (15–24 years) and adults (25–64 years) increased over time, while the elderly (65 + years) also showed an increase, from 7.05% in 2009/2010 to 11.93% in 2019/2020.

The geographic distribution across regions indicated a higher proportion of individuals in the Southern Region compared to the Northern Region in all waves, with the Southern Region showing an increase from 70.13% in 2009/2010 to 78.15% in 2019/2020. Regarding marital status, the proportion of those who were married or in a consensual union decreased over time, while the percentage of individuals who had never married increased substantially, reaching 57.32% in 2019/2020.

Religious affiliation was predominantly Christian, followed by Muslim and Traditional religions across the survey waves. Employment status showed a consistently high unemployment rate, with minor variations over time. The educational level data indicates a relatively stable percentage of individuals with no formal education, while the proportion of individuals with primary and secondary education increased slightly.

Depression prevalence varied over time, with most respondents experiencing no significant depression in each wave. Health-seeking behavior among individuals with depressive symptoms showed an increasing trend, with the percentage of those seeking consultation for depression rising from 6.38% in 2009/2010 to 14.88% in 2019/2020.

**Table 1:** Description of socio-economic variables across three waves.

Variables	Waves					
	2009/2010		2013/2014		2019/2020	
	Freq.	Percent	Freq.	Percent	Freq.	Percent
Gender						
Female	9816	52.42	8121	52.27	6547	53.00
Male	8910	47.58	7417	47.73	5806	47.00
Age						
Children (0–14)	7516	40.13	5838	36.08	7427	33.70
Youth (15–24)	3098	16.54	2461	15.21	3789	17.19
Adult (25–64)	6795	36.28	6030	37.27	8192	37.17
Elderly (65 and above)	1320	7.05	1852	11.45	2629	11.93
Region						
Southern Region	13,135	70.13	11,161	68.98	16,563	78.15
Northern Region	5594	29.87	5019	31.02	4630	21.85
Marital status						
Married/Consensual Union	6258	49.68	5182	33.40	6771	31.95
Separated/Divorced	685	5.44	681	4.39	947	4.47
Widowed	857	6.80	907	5.85	1327	6.26
Never married	4796	38.08	8,747	56.37	12,148	57.32
Religion						
None	791	4.34	653	6.07	817	3.86
Christian	12,090	66.38	7300	67.83	14,658	69.25
Muslim	3731	20.48	2192	20.37	4507	21.29
Traditional	1581	8.68	595	5.53	1089	5.14
Others	21	0.12	22	0.20	96	0.45
Employment status						
Employed	2164	12.55	1,112	7.16	2774	13.16
Unemployed	15,077	87.45	14,426	92.84	18,298	86.84
Educational level						
None	7673	64.46	7820	67.05	9030	65.73
Primary	1350	11.34	2563	21.98	3076	22.39
Secondary	2489	20.91	922	7.91	1161	8.45
Post-secondary	391	3.28	358	3.07	471	3.43
Communal engagement						
Yes	7252	39.24	4166	26.83	5151	27.29
No	11,227	60.76	11,361	73.17	13,726	72.71
Depression						
No significant depression	12,879	68.96	11,302	72.79	13,259	70.46
Mild depression	3492	18.70	2706	17.43	3153	16.76
Moderate depression	1645	8.81	1099	7.08	1745	9.27
Severe depression	661	3.54	420	2.70	661	3.51
Consultation for depression						
Yes	1157	6.38	1838	11.84	2274	14.88
No	16,968	93.62	13,689	88.16	13,010	85.12

### Regression analyses of depression status

Table 2 presents the findings from logistic regression analyses examining the effect of socioeconomic factors on depression status across survey waves and in the combined full sample. The odds ratios (ORs) facilitate interpretation of the probability of experiencing depression associated with each predictor.

For gender, the odds ratios for females were not statistically significant across all survey waves, suggesting that gender did not have a meaningful effect on depression status. Age, however, showed a strong effect on depression likelihood, particularly among older age groups. The elderly (65+) consistently exhibited higher odds of depression in all survey waves, with ORs of 1.647 (*p* < 0.01) in 2009/2010, 2.027 (*p* < 0.01) in 2013/2014, and 1.691 (*p* < 0.05) in 2019/2020. Adults also displayed elevated odds, particularly in the full sample (OR = 1.170, *p* < 0.05).

Region of residence demonstrated a significant effect, with individuals living in the Northern Region having consistently higher odds of depression across all survey periods. This effect was especially prominent in 2009/2010 (OR = 2.103, *p* < 0.01), with elevated odds persisting in later waves and in the full sample (OR = 1.814, *p* < 0.01). Household size had a modest but significant protective effect against depression, particularly in the full sample, where larger households were associated with slightly reduced odds of depression (OR = 0.983, *p* < 0.05).

Marital status showed varied effects across categories. Being married or in a consensual union was generally associated with lower odds of depression, with ORs of 0.793 (*p* < 0.1) in 2009/2010 and 0.725 (*p* < 0.01) in the full sample. Conversely, widowed individuals had significantly higher odds of depression in 2019/2020 (OR = 1.820, *p* < 0.05). Religious affiliation also had an effect, particularly among individuals identifying with traditional beliefs; in the full sample, traditional religious affiliation was associated with increased odds of depression (OR = 1.380, *p* < 0.05).

**Table 2:** Results from logistic regression on effect of socio-economic variables to depression status.

Variables	Depression status			
	2009/2010	2013/2014	2019/2020	Full sample
Gender				
Female	1.035	1.033	0.938	1.015
	(0.0574)	(0.0573)	(0.0631)	(0.0352)
Age				
Youth (15–24)	1.006	1.255**	0.832**	0.923
	(0.0976)	(0.0934)	(0.0934)	(0.0519)
Adults (25–64)	1.255*	1.471***	1.517***	1.170**
	(0.129)	(0.128)	(0.147)	(0.0760)
Elderly (65+)	1.647***	2.027***	1.691**	1.531***
	(0.166)	(0.176)	(0.238)	(0.111)
Region				
Northern regions	2.103***	2.030***	1.326***	1.814***
	(0.0832)	(0.0792)	(0.0769)	(0.0610)
Household size	1.004	0.980	0.955***	0.983**
	(0.0123)	(0.0124)	(0.0145)	(0.00767)
Marital status				
Married/Consensual Union	0.793*	0.606***	1.179	0.725***
	(0.119)	(0.109)	(0.215)	(0.0808)
Widowed	1.029	1.003	1.820**	1.093
	(0.157)	(0.153)	(0.274)	(0.106)
Never married	0.936	0.791*	1.843**	0.932
	(0.151)	(0.135)	(0.249)	(0.0966)
Religion				
Christians	1.100	0.735**	1.157	0.912
	(0.143)	(0.120)	(0.180)	(0.0850)
Muslim	1.068	0.874	1.517**	1.128
	(0.160)	(0.137)	(0.190)	(0.0934)
Traditional	1.462**	1.060	1.434	1.380**
	(0.190)	(0.219)	(0.262)	(0.128)
Employment status				
No	0.568***	0.807**	0.795**	0.674***
	(0.0825)	(0.0930)	(0.0965)	(0.0593)
Educational level				
Primary	0.917	0.866**	0.861*	0.882***
	(0.0893)	(0.0658)	(0.0844)	(0.0461)
Secondary	0.825***	0.688***	0.836	0.723***
	(0.0733)	(0.102)	(0.135)	(0.0589)
Post-secondary	0.598**	0.600***	0.873	0.597***
	(0.213)	(0.162)	(0.200)	(0.114)
Communal engagement				
No	0.792***	0.695***	0.747***	0.752***
	(0.0568)	(0.0601)	(0.0680)	(0.0414)
Income	0.999742***	0.999969***	0.999941***	0.999951***
	(3.49e−05)	(1.01e−05)	(1.07e−05)	(7.16e−06)
Constant	0.626**	0.718	0.414***	0.713**
	(0.238)	(0.218)	(0.331)	(0.148)
Pseudo R^2^				
Observations	7315	7654	5290	20,269

The table presents odds ratios (OR) for each variable, with Standard errors in parentheses, *** *p* < 0.01, ** *p* < 0.05, * *p* < 0.1. The results in columns 2–4 reflect wave-specific cross-sectional logit regressions estimated separately for the 2009/2010, 2013/2014, and 2019/2020 survey waves. The final column (full sample) presents results from a random-effects panel logit model using all three waves combined, accounting for time-invariant individual characteristics.

Employment status, particularly unemployment, was consistently linked with lower odds of depression across all survey waves, with significant effects seen in 2009/2010 (OR = 0.568, *p* < 0.01) and the full sample (OR = 0.674, *p* < 0.01). Educational attainment demonstrated a protective effect against depression, as higher education levels, particularly secondary and post-secondary education, were associated with lower odds of depression. In the full sample, individuals with secondary education had an OR of 0.723 (*p* < 0.01), while those with post-secondary education had an OR of 0.597 (*p* < 0.01). Communal engagement was also a significant factor, with a lack of engagement linked to lower odds of depression across all waves; in the full sample, non-participation in communal activities had an OR of 0.752 (*p* < 0.01). Finally, income showed a very small but statistically significant protective effect across all waves and the full sample, with ORs close to 1 (OR = 0.999951, *p* < 0.01 in the full sample). This indicates that for each GHC1 increase in income, the odds of depression decrease slightly.

### Regression analyses of health-seeking behavior

Table 3 presents the results from logistic regression analysis on health-seeking behavior among individuals with depressive symptoms. The odds ratios reveal factors associated with the likelihood of seeking healthcare for depression.

Gender did not have a statistically significant effect across the survey waves. Age was not a strong predictor of health-seeking behavior, though the youth group (15–24 years) showed lower odds in the full sample (OR = 0.668). Residing in the Northern Region was consistently associated with lower odds of seeking healthcare for depression, with ORs of 0.496 in 2009/2010 and 0.364 in the full sample. Larger household size was linked to a reduced likelihood of seeking care in 2019/2020 (OR = 0.891) and in the full sample (OR = 0.837).

Marital status showed varied associations, with widowed individuals in 2013/2014 having lower odds (OR = 0.601). Religion did not show significant associations in the full sample, though some variation occurred across waves. Employment status was not significantly associated with health-seeking behavior across waves. Secondary education was linked to lower odds of seeking healthcare in 2009/2010 (OR = 0.634) and in the full sample (OR = 0.398). Lack of communal engagement was associated with lower odds of seeking healthcare in 2013/2014 (OR = 0.796). Income was positively associated with health-seeking behavior in 2019/2020 (OR = 1.000) and in the full sample (OR = 1.000).

**Table 3:** Logistic regression results on the effect of socioeconomic variables on Health-Seeking behavior among depressed individuals across survey Waves.

Variables	Health-seeking behavior			
	2009/2010	2013/2014	2019/2020	Full sample
Gender				
Female	0.900	1.186	0.933	0.993
	(0.139)	(0.142)	(0.118)	(0.233)
Age				
Youth (15–24)	0.877	0.907	1.086	0.668
	(0.237)	(0.186)	(0.206)	(0.232)
Adults (25–64)	0.555	1.032	0.953	0.566
	(0.205)	(0.272)	(0.253)	(0.278)
Elderly (65+)	1.060	1.530	0.889	1.459
	(0.461)	(0.533)	(0.399)	(0.990)
Region				
Northern regions	0.496***	0.550***	0.749*	0.364***
	(0.121)	(0.096)	(0.115)	(0.110)
Household size	0.964	0.995	0.891***	0.837***
	(0.032)	(0.025)	(0.028)	(0.043)
Marital status				
Married/Consensual Union	1.264	0.951	0.645	0.919
	(0.411)	(0.193)	(0.266)	(0.453)
Widowed	1.381	0.601*	1.192	0.877
	(0.542)	(0.177)	(0.593)	(0.568)
Never married	0.679	0.930	0.702	0.728
	(0.296)	(0.243)	(0.325)	(0.451)
Religion				
Christians	0.950	0.940	2.061	1.167
	(0.356)	(0.220)	(0.930)	(0.653)
Muslim	1.429	0.867	1.767	1.120
	(0.602)	(0.243)	(0.829)	(0.693)
Traditional	0.727	0.813	0.595	0.286
	(0.379)	(0.353)	(0.449)	(0.257)
Employment status				
No	0.786	0.973	1.322	1.223
	(0.156)	(0.181)	(0.266)	(0.425)
Educational level				
Primary	0.714	1.003	0.838	0.949
	(0.176)	(0.134)	(0.141)	(0.281)
Secondary	0.634**	0.916	1.104	0.398**
	(0.134)	(0.197)	(0.289)	(0.154)
Post-secondary	1.148	1.343	1.396	3.240
	(0.587)	(0.419)	(0.518)	(2.353)
Communal engagement				
No	0.847	0.796*	0.851	0.686
	(0.129)	(0.096)	(0.112)	(0.163)
Income	1.000	1.000*	1.000***	1.000***
	(0.000)	(0.000)	(0.000)	(0.000)
Constant	0.328*	0.405**	0.340	0.000***
	(0.210)	(0.175)	(0.235)	(0.000)
Pseudo R^2^	0.0376	0.0257	0.0334	0.0494
Observations	1811	1854	1429	5094

The table presents odds ratios (OR) for each variable, with Standard errors in parentheses, *** *p* < 0.01, ** *p* < 0.05, * *p* < 0.1. The results in columns 2–4 reflect wave-specific cross-sectional logit regressions estimated separately for the 2009/2010, 2013/2014, and 2019/2020 survey waves. The final column (full sample) presents results from a random-effects panel logit model using all three waves combined, accounting for time-invariant individual characteristics. Income was positively associated with health-seeking behavior, though the odds ratios appear small (OR ≈ 1.000). This means that for every one-cedi increase, the change in odds is minimal; however, over larger amounts, the effect becomes meaningful. For example, a 1000-cedi increase raises the odds of seeking healthcare by about 10%, highlighting how household income differences shape access to mental health services.

## Sensitivity analyses

The sensitivity analyses, as shown in Table 4, reveal important insights into how socio-economic factors relate to depression scores when assessed as a continuous variable. Age remained a significant predictor, with elderly individuals (65+) consistently exhibiting the highest depression scores across all waves and the full sample. This aligns with the baseline analysis, where older adults demonstrated higher odds of depression. Regional disparities persisted, as residents of the Northern Region consistently showed higher depression scores, mirroring the baseline findings of elevated odds of depression in this region.

Household size had a protective effect, with larger households associated with slightly lower depression scores, consistent with the baseline analysis where household size reduced the odds of depression. Marital status also showed comparable patterns, with married individuals experiencing lower depression scores and widowed individuals showing increased vulnerability. Similarly, higher education levels and communal engagement were negatively associated with depression scores, paralleling the protective effects observed in the binary analysis. Income consistently showed a statistically significant negative association with depression scores across all waves and in the full sample. Although the coefficients (–0.000309 in 2009/2010, − 7.63e−06 in 2013/2014, − 1.30e−05 in 2019/2020, and − 0.000617 in the full sample) appear numerically small, they indicate that even slight increases in income are linked to measurable reductions in depression scores.

**Table 4:** Robustness check Results – Effects of Socio-Economic variables on depression scores across Waves.

Variables	Depression scores			
	2009/2010	2013/2014	2019/2020	Full sample
Gender				
Female	0.0399	0.0227	0.0290	0.333***
	(0.128)	(0.0213)	(0.0258)	(0.103)
Age				
Youth (15–24)	0.228	0.110***	− 0.0274	2.081***
	(0.214)	(0.0348)	(0.0370)	(0.152)
Adults (25–64)	0.491*	0.245***	0.183***	2.205***
	(0.287)	(0.0480)	(0.0621)	(0.223)
Elderly (65+)	1.220***	0.429***	0.203**	3.870***
	(0.380)	(0.0680)	(0.100)	(0.324)
Region				
Northern regions	2.272***	0.229***	0.104***	0.323**
	(0.199)	(0.0313)	(0.0323)	(0.140)
Household size	− 0.0387	− 0.0184***	− 0.0229***	0.146***
	(0.0279)	(0.00469)	(0.00574)	(0.0224)
Marital status				
Married/Consensual Union	− 0.819***	− 0.209***	0.116	0.0942
	(0.277)	(0.0434)	(0.0860)	(0.238)
Widowed	− 0.0303	0.00331	0.203*	0.497
	(0.376)	(0.0628)	(0.116)	(0.332)
Never married	− 0.656*	− 0.0723	0.266***	− 0.973***
	(0.348)	(0.0532)	(0.101)	(0.293)
Religion				
Christians	0.189	− 0.0917*	0.139**	1.056***
	(0.318)	(0.0482)	(0.0707)	(0.255)
Muslim	0.580	− 0.0663	0.110	0.884***
	(0.362)	(0.0551)	(0.0753)	(0.282)
Traditional	1.367***	0.117	0.153	3.649***
	(0.452)	(0.0910)	(0.108)	(0.399)
Others	− 1.117	− 0.191	0.0455	− 1.432
	(1.699)	(0.242)	(0.184)	(1.010)
Employment status				
No	− 1.289***	− 0.0783**	− 0.0518	0.0441
	(0.203)	(0.0346)	(0.0403)	(0.162)
Educational level				
Primary	− 0.407**	− 0.0387	− 0.0200	− 2.980***
	(0.197)	(0.0249)	(0.0343)	(0.132)
Secondary	− 0.631***	− 0.0559	− 0.146***	2.326***
	(0.158)	(0.0364)	(0.0536)	(0.156)
Post-secondary	− 0.936**	− 0.0891	− 0.237***	− 0.251
	(0.391)	(0.0560)	(0.0789)	(0.293)
Communal engagement				
No	− 0.932***	− 0.100***	− 0.236***	− 2.140***
	(0.127)	(0.0230)	(0.0286)	(0.108)
Income	− 0.000309***	− 7.63e−06***	− 1.30e−05***	− 0.000617***
	(4.63e−05)	(2.86e−06)	(3.33e−06)	(1.54e−05)
Constant	18.56***	1.852***	1.739***	6.956***
	(0.543)	(0.0849)	(0.134)	(0.439)
Observations	7325	7654	5302	20281
R-squared	0.066	0.031	0.032	0.174

Standard errors in parentheses, *** *p* < 0.01, ** *p* < 0.05, * *p* < 0.1. The results in columns 2–4 reflect wave-specific cross-sectional logit regressions estimated separately for the 2009/2010, 2013/2014, and 2019/2020 survey waves. The final column (full sample) presents results from a random-effects panel logit model using all three waves combined, accounting for time-invariant individual characteristics.

## Discussion

This study provides valuable insights into the prevalence of depression, health-seeking behavior, and the influence of socioeconomic factors among Ghanaians over a 10-year period. Logistic regression analysis highlights several consistent predictors of depression and health-seeking behavior, emphasizing vulnerable groups and the need for targeted mental health interventions.

The study confirms that age significantly influences depression likelihood, particularly among elderly individuals. Older adults face heightened mental health vulnerability due to isolation, reduced social support, and physical health challenges^65^. These findings align with global trends, including Banerjee et al.^66^, who observed higher depression prevalence among individuals aged 55 and above in LMICs compared to higher-income countries. Similarly, Brinda et al.^67^ found that aging and socioeconomic adversity exacerbate depression risk in resource-limited settings, highlighting the need for targeted interventions for elderly populations.

Region of residence emerged as a critical determinant, with residents of the Northern Region showing consistently higher odds of depression. This regional disparity parallels patterns observed in other contexts, where limited mental health infrastructure and socioeconomic disadvantages exacerbate mental health outcomes^68^. For instance, Rajper et al.^69^ observed similar disparities in Pakistan, with urban-related stressors and economic disparities affecting mental health outcomes. In Ghana’s Northern Region, limited healthcare access and economic challenges compound these effects, requiring policy attention to address the unique needs of these regions.

Marital status also influences depression, with married individuals exhibiting lower odds of depression compared to their widowed counterparts. These findings align with Zhao et al.^70^ and Hsu et al.^71^, who highlighted the protective role of social bonds in mitigating mental health risks. Addressing the mental health needs of widowed or separated individuals, particularly in resource-limited settings, could significantly improve mental health outcomes. Higher education is a protective factor against depression, with educated individuals displaying lower depressive symptoms and better health-seeking behaviour. This is consistent with studies by Gudjonsson et al.^72^ and Tassone et al.^73^, which emphasize the role of education in enhancing mental health literacy and reducing stigma.

The unique finding that unemployed individuals in Ghana exhibited lower odds of depression challenges typical associations between unemployment and poorer mental health. This result may reflect cultural factors in Ghana, where extended family networks and community structures provide substantial social and financial support to unemployed individuals, buffering the adverse mental health effects of unemployment^74^. Such informal support systems are deeply rooted in Ghanaian traditions, where communal living and familial interdependence play a pivotal role in individual well-being Daliri et al.^75^. Van Oosten et al.^76^ and Sambasivam et al.^50^ emphasized the role of social support in mental health resilience, and these findings highlight the importance of considering socio-cultural contexts when analyzing unemployment’s impact on mental health. However, it is essential to investigate whether these support systems can sustain resilience in the face of increasing urbanization and weakening traditional social networks in Ghana.

Regional disparities in depression prevalence underscore the impact of structural inequalities and cultural norms in Ghana. In particular, the Northern Region consistently showed higher odds of depression, reflecting broader patterns of economic and infrastructural disparities. These findings align with Tsimpida et al.^77^, who linked regional inequalities in England to worsening depression trends in socioeconomically deprived areas, and Aretz^78^, who highlighted how economic crises exacerbated mental health risks in disadvantaged regions. In Ghana, the concentration of healthcare infrastructure, resources, and mental health professionals in urban areas, particularly in Accra and Kumasi, creates barriers for rural and underserved regions^6^. The Northern Region’s limited access to healthcare facilities, coupled with lower literacy rates and higher poverty levels, exacerbates mental health challenges^79^.

The protective role of education in mitigating depression risk and fostering health-seeking behavior is notable across contexts. In Ghana, education not only enhances mental health literacy but also reduces stigma, empowering individuals to recognize and address mental health issues^80^. For instance, Mumulati et al.^81^ found that educated elderly individuals were more likely to seek mental health services, reducing stigma and improving outcomes. Similarly, education in Ghana plays a critical role in raising awareness about available mental health resources and encouraging timely intervention. However, disparities in educational access, especially in rural areas, continue to hinder the potential benefits of education on mental health outcomes^82^.

Although the income coefficients in our results appear small in absolute terms, their cumulative impact is meaningful. A GHS 1,000 increase in household income, approximately USD 83, is associated with a 0.6-point reduction in depression scores, which is notable given that the K10 ranges from 10 to 50. This aligns with prior studies demonstrating the relationship between financial stress, income, and mental health. Guan et al.^83^, through a systematic review of 40 observational studies, confirmed that financial stress is positively associated with depression in both high-income and low- and middle-income countries, with stronger effects observed among low-income populations. They highlighted pathways such as social causation, psychological stress, and social selection in explaining how financial hardship fuels depression. Similarly, Li et al.^84^, using large-scale Chinese data, showed that income increases significantly reduce depression risk at lower-income levels, reinforcing the value of economic interventions for vulnerable households.

Some limitations must be acknowledged. First, reliance on self-reported measures of depression and health-seeking behavior may introduce bias, including underreporting or recall errors. Second, the use of random-effects modelling assumes unobserved heterogeneity is uncorrelated with predictors, which, if untrue, could bias the results. Third, the inability to estimate unit fixed effects due to non-varying outcomes across waves limits the ability to fully account for individual-specific characteristics.

## Implications for policy and research

This study highlights the urgent need for targeted mental health policies and interventions in Ghana. Expanding mental health infrastructure in underserved regions like the Northern Region is critical to addressing regional disparities. Integrating mental health services into primary healthcare and decentralizing care delivery can improve access, particularly in rural areas.

Educational initiatives promoting mental health literacy should be prioritized to reduce stigma and enhance health-seeking behaviour. Policies aimed at strengthening social support systems, particularly for widowed or separated individuals, are essential for mitigating mental health risks. Culturally sensitive approaches are also needed to address the unique challenges associated with unemployment and regional inequalities in Ghana.

Future research should explore the interaction of cultural, socioeconomic, and environmental factors in shaping mental health outcomes.

## Conclusion

This study analyzed depression prevalence and health-seeking behavior in Ghana over a decade, highlighting key socio-economic factors. Depression was notably high among the elderly and residents of the Northern Region, with age, marital status, education, and communal engagement identified as significant predictors. Older adults and widowed individuals faced higher depression risks, while education and social engagement offered protective effects. Despite improvements in health-seeking behavior, access remained limited for rural and disadvantaged groups. Recommendations included expanding mental health services for the elderly, addressing regional disparities, supporting widowed individuals, enhancing mental health education in schools, and fostering community engagement programs.

## Electronic supplementary material

Below is the link to the electronic supplementary material.

Supplementary Material 1

## Data Availability

The datasets used in this article are available via the Institute of Statistical, Social and Economic Research data repository < https://dataportal-isser.ug.edu.gh/index.php/catalog/4> and can be requested on the website.
