# Supplementary material for: Estimating the prevalence, socioeconomic determinants, and health seeking behavior of individuals with depression in Ghana
Source: Sci Rep. 2025 Jul 1;15:22239. doi: 10.1038/s41598-025-06134-2 (PMC12215751; doi:10.1038/s41598-025-06134-2)
Supplement: Supplementary file 1 — Supplementary Material 1 [file 41598_2025_6134_MOESM1_ESM.pdf]

Table S1: Kessler 10 Psychological Distress Scale (K10) Survey Questions

Question Number	Question
A1	About how often did you feel tired out for no good reason?
A2	About how often did you feel nervous?
A3	About how often did you feel so nervous that nothing could calm you down?
A4	About how often did you feel hopeless?
A5	About how often did you feel restless or fidgety?
A6	About how often did you feel so restless you could not sit still?
A7	About how often did you feel depressed?
A8	About how often did you feel that everything was an effort?
A9	About how often did you feel so sad that nothing could cheer you up?
A10	About how often did you feel worthless?

Note: Response Options for All Questions: 1. None of the time, 2. A little of the time, 3. Some of the time, 4. Most of the time and 5. All of the time.

Table S2: Socio-Economic Variables, Definitions, and Measurement

Variable	Definition	Measurement Categories
**Gender**	Biological sex of the respondent	Female, Male
**Age**	Age groups reflecting different life stages	Children (0-14), Youth (15-24), Adults (25-64), Elderly (65+)
**Region**	Geographic division of respondents	Southern Region, Northern Region
**Marital Status**	Current marital or relationship status	Married/Consensual Union, Separated/Divorced, Widowed, Never Married
**Religion**	Religious affiliation of the respondent	None, Christian, Muslim, Traditional, Others
**Employment Status**	Current employment status	Employed, Unemployed
**Educational Level**	Highest level of education completed	None, Primary, Secondary, Post-Secondary
**Communal Engagement**	Participation in community-based activities	Yes, No
